# Toward the elimination of hepatitis B: networking to promote the prevention of vertical transmission of hepatitis B virus through population-based interventions and multidisciplinary groups in Africa

**DOI:** 10.3389/fpubh.2024.1283350

**Published:** 2024-04-05

**Authors:** Judith Ndongo Embola Torimiro, Kerina Duri, Nadège M. Goumkwa, Solange M. Atah, Juliette-Laure Ndzie Ondigui, Cindy Lobe, Marielle Bouyou, Bénédicte Ndeboko, Ali Mahamat Moussa, Camengo Police, Patrick Awoumou, Puinta Peyonga, Prisca V. Djivida, Assah Felix, Godwin W. Nchinda, Brigitte Wandji, Rachel K. Simo, Sylvie Agnès Moudourou, Ana Gutierrez, Rosi Garcia, Isabelle Fernandez, Evelyn Mah, Sarah Rowland-Jones, Robinson Mbu

**Affiliations:** ^1^Molecular Biology Laboratory, Chantal BIYA International Reference Center for Research on HIV/AIDS Prevention and Management (CIRCB), Yaoundé, Cameroon; ^2^Department of Biochemistry, Faculty of Medicine and Biomedical Sciences, University of Yaoundé I, Yaoundé, Cameroon; ^3^Department of Immunology, College of Health Sciences, University of Zimbabwe, Harare, Zimbabwe; ^4^Department of Public Health, Faculty of Medicine and Biomedical Sciences, University of Yaoundé I, Yaoundé, Cameroon; ^5^Department of Microbiology, Faculty of Science, University of Yaoundé I, Yaoundé, Cameroon; ^6^Department of Food Science and Nutrition, University of Ngaoundere, Ngaoundere, Cameroon; ^7^Department of Parasitology, Mycology and Tropical Medicine, University of Health Sciences, Libreville, Gabon; ^8^Department of Cell and Molecular Biology-Genetics, Faculty of Medicine, University of Health Sciences, Libreville, Gabon; ^9^Centre Hospitalier Universitaire Mère-Enfant de la Fondation Jeanne EBORI (CHUMEFJE), Libreville, Gabon; ^10^Gastroenterology and Internal Medicine Unit, University Reference Hospital, Gamena, Chad; ^11^Department of Hepato-Gastroenterology and Internal Medicine of “Amitié Sino Centrafraine”, University Hospital Center, Bangui, Central African Republic; ^12^Vaccinology Laboratory, Chantal BIYA International Reference Center for Research on HIV/AIDS Prevention and Management (CIRCB), Yaoundé, Cameroon; ^13^Yaoundé Gynaecology, Obstetrics and Pediatrics Hospital, Yaoundé, Cameroon; ^14^Clinical Diagnostic Laboratory, Chantal BIYA International Reference Center for Research on HIV/AIDS Prevention and Management (CIRCB), Yaoundé, Cameroon; ^15^Medical Unit, Chantal BIYA International Reference Center for Research on HIV/AIDS Prevention and Management (CIRCB), Yaoundé, Cameroon; ^16^Bikop Catholic Health Center, Bikop, Cameroon; ^17^Department of Paediatrics, Faculty of Medicine and Biomedical Sciences, University of Yaoundé I, Yaoundé, Cameroon; ^18^Nuffield Department of Medicine, Medical Sciences Division, University of Oxford, Oxford, United Kingdom; ^19^Department of Gynaecology and Obstetrics, Faculty of Medicine and Biomedical Sciences, University of Yaoundé I, Yaoundé, Cameroon

**Keywords:** hepatitis B, birth-dose, vaccine, vertical, transmission, prevention, Africa

## Abstract

The WHO African Region had 81 million people with chronic hepatitis B in 2019, which remains a silent killer. Hepatitis B virus (HBV), hepatitis delta virus (HDV), and HIV can be transmitted from the mother to child. If the HBV infection is acquired at infancy, it may lead to chronic hepatitis B in 90% of the cases. WHO reports that 6.4 million children under 5 years live with chronic hepatitis B infection worldwide. The prevention of mother-to-child transmission (PMTCT) of HBV is therefore critical in the global elimination strategy of viral hepatitis as we take lessons from PMTCT of HIV programs in Africa. We sought to create a network of multidisciplinary professional and civil society volunteers with the vision to promote cost-effective, country-driven initiatives to prevent the MTCT of HBV in Africa. In 2018, the Mother–Infant Cohort Hepatitis B Network (MICHep B Network) with members from Cameroon, Zimbabwe, and the United Kingdom and later from Chad, Gabon, and Central African Republic was created. The long-term objectives of the network are to organize capacity-building and networking workshops, create awareness among pregnant women, their partners, and the community, promote the operational research on MTCT of HBV, and extend the network activities to other African countries. The Network organized in Cameroon, two “Knowledge, Attitude and Practice” (KAP) surveys, one in-depth interview of 45 health care workers which revealed a high acceptability of the hepatitis B vaccine by families, two in-person workshops in 2018 and 2019, and one virtual in 2021 with over 190 participants, as well as two workshops on grant writing, bioethics, and biostatistics of 30 postgraduate students. Two HBV seroprevalence studies in pregnant women were conducted in Cameroon and Zimbabwe, in which a prevalence of 5.8% and 2.7%, respectively, was reported. The results and recommendations from the MICHep B Network activities could be implemented in countries of the MICHep B Network and beyond, with the goal of providing free birth dose vaccine against hepatitis B in Africa.

## 1 Introduction

Hepatitis B is vaccine-preventable (1), sexually transmissible, and caused by the hepatitis B virus (HBV), which is blood-borne and can be transmitted from the mother to child. It is considered a silent killer in Africa affecting 81 million (27.36%) people who live with chronic hepatitis B out of 296 million cases globally in 2019 (1–3). In 2016, WHO recommended goals to reduce hepatitis B-related incidence by 90% and its mortality by 65% by 2030 (4) and reported that 6.4 million children live with chronic hepatitis B worldwide. HBV infection acquired at birth or in the early years of life may lead to chronic infection and an increased risk of developing liver complications and death in adulthood (5). WHO promotes the prevention of mother-to-child transmission (PMTCT) of HBV as a foundational pillar in the global elimination strategy of viral hepatitis (6). Establishing and scaling up interventions to reach pregnant woman, screen them, sensitize and screen their partners, and eventually refer them to care or vaccination for hepatitis B have been slow in Cameroon, Zimbabwe, Central African Republic, Chad, and Gabon. A few studies of HBV seroprevalence carried out among pregnant women from 1991 to 2019 in Cameroon, Central African Republic, Chad, Gabon, and Zimbabwe, show prevalence of 6.4%, 8.2%, 25%, 9.2%, and 13.0%, respectively, without taking into consideration the rates of seroconversion and occult hepatitis B infection during the index pregnancy (7–13). Although the rate of vertical transmission of HBV in these African countries is not known, Keane et al. reported in a review article from 11 sub-Saharan African (SSA) countries that the pooled risk without prophylaxis was 38.3% among HBeAg-positive pregnant women, 4.8% among HBeAg-negative women, and an estimated 1% of newborns who are annually infected at birth because of infectious mothers (14). Nonetheless, it is worth noting that community-based organizations and groups have successfully implemented sensitization and prevention strategies in Africa to control the spread of HIV/AIDS. The Society for Women and AIDS in Africa (SWAA), for instance, created in 1988 by African women, advocates for education of women to bring about a positive change and prevent the disease (15).

Without preventive interventions such as newborn postexposure prophylaxis (PEP) of the birth-dose vaccine and/or hepatitis B immunoglobulin (HBIG) given within 24 h of life and/or paripartum maternal antiviral prophylaxis (MAP), mother-to-child transmission (MTCT) of HBV will remain the primary route of infection in Africa (4, 16–18). In 2019, the global rate of HepB-BD vaccine coverage was 44% compared to 42% in 2021 (19). In 2021, 14 out of 47 countries in Africa had incorporated HepB-BD vaccine in the Expanded Programme on Immunization (EPI) with 17% of the children (compared to 10% in 2017) receiving timely HepB-BD vaccine (20). However, most sub-Saharan African (SSA) governments and the Global Alliance Vaccine Initiative (GAVI) do not subsidize the HepB-BD vaccine. By the end of 2022, none of the countries in the MICHep B Network had HepB-BD vaccine in their EPI, but Cameroon added the vaccine into its EPI in January 2023. The global three-dose vaccine coverage for hepatitis B was 71% in 2021, which was below the 90% target (21). However, families and the communities show a great interest in contributing to the fight against hepatitis B.

We sought to create a network of a multidisciplinary team from Cameroon, Central African Republic, Chad, Gabon, and Zimbabwe to promote innovative, cost-effective, country-driven, accessible, add-on initiatives to reduce the MTCT of HBV and build on the experience of the countries' PMTCT of HIV and Syphilis Programmes. The objectives of the network are to organize capacity-building and training workshops conducted by multidisciplinary experts on PMTCT of HBV, create awareness among pregnant women, their partners, and the community on PMTCT of HBV, extend the network activities to other African countries, and promote research on antiviral prophylaxis in PMTCT of HBV and co-infections with HIV.

## 2 Materials and methods

### 2.1 Study design

A mixed-methods design with qualitative and quantitative survey methods were employed in a cross-sectional and observational study to collect data. The mixed survey methods included in-depth interviews, creation of networks and expansion to other countries, capacity building of young researchers, sensitization, and serosurveys. A purposive sampling method was used to select the countries in the MICHep B Network in the Central African sub-Region, while a convenient sampling method was used to choose the health facilities for the serosurvey. The selected countries in the Central African sub-Region were high-endemic hepatitis B countries and without a national hepatitis B PMTCT program. In addition, low awareness of the disease among health care workers and the general population in these countries is low. Zimbabwe and Cameroon submitted a joint proposal for funding, which was accepted for a serosurvey of hepatitis B among pregnant women in both countries with the goal of setting up a network. The partner in North Africa was selected because of the ongoing collaboration between Cameroon and Zimbabwe, and a common interest in mobilizing funds from the Academy of Medical Sciences in the UK to support the implementation of the MICHep B Network activities.

### 2.2 Data collection

#### 2.2.1 Study sites

The study site in the Center Region of Cameroon was selected because of a high seroprevalence of HBsAg of 9.1% and early sexual activity in the population, specifically by the age of 14 years (22). In the main study, one general hospital, four district hospitals, and one health center in Cameroon and one tertiary hospital in Zimbabwe were selected using a convenient sampling method, and the sampling was done from 2018 to 2022.

#### 2.2.2 In-depth interviews

A purposive sampling method was used to select participants for the in-depth interviews in Cameroon. The interviews were conducted among health care providers (physicians, nurses, and midwives) and senior policymakers of the Family Health Department of the Ministry of Health and professional associations, including the Society of Gynecologists and Obstetricians of Cameroon (SOGOC), Cameroon Society of Pediatrics (SOCAPED), and Cameroon Association of Gastroenterology (SCGE). The interview questions were geared toward guidelines, policy, and practice of PMTCT of HBV, community involvement in PMTCT activities, field experience, and challenges.

#### 2.2.3 Population-based sensitization and HBV seroprevalence study in Cameroon and Zimbabwe

A community-based sensitization on hepatitis B was conducted before the two pilot seroprevalence studies of HBV infection were carried out among pregnant women in Yaoundé (Cameroon) and Harare (Zimbabwe). Following informed consent, the pregnant women filled questionnaires, and they were later tested for HBsAg using a rapid diagnostic test (RDT) and enzyme-linked immunosorbent assay (ELISA). The women were notified of their results at the hospital within 24 h for the non-reactive cases and within 2 weeks for the positive cases. Those who were negative for HBsAg were advised to get vaccinated against hepatitis B and to encourage other individuals of their household to get tested voluntarily. The positive cases were enrolled into the cohort for follow-up and to ensure the acquisition and timely administration of the hepatitis B birth-dose vaccine for the newborns.

#### 2.2.4 Creation and expansion of the network and identification of country focal points

The first meeting was held in October 2018 in Yaoundé, Cameroon, between the key players from Cameroon, Zimbabwe, and the United Kingdom, which included a principal investigator and staff of the Family Health Department of the Cameroon Ministry of Health to identify the activities, research goals, countries, and focal points of the “Mother–Infant Cohort of Hepatitis B Network” (MICHep B Network) (Supplementary material 1). A training workshop on grant writing, bioethics, and biostatistics, and “How to search for funding agencies for research” was organized in 2018. Extensive literature review on PMTCT strategies of hepatitis B in the member countries of the MICHep B Network was performed using Google Scholar, PubMed, and WHO recommendations to guide the development of the Work Plan for the Network (Supplementary material 2).

#### 2.2.5 Organization of capacity-building, training, and networking workshops

Capacity-building and networking workshops were held in-person in 2018 and 2019 for multidisciplinary groups of health professionals including gynecologists, pediatricians, researchers, public health experts, high-ranking Ministry of Health personnel, university lecturers, and students from Cameroon, Chad, Gabon, Zimbabwe, United Kingdom, and the United States, not leaving out the civil society. In 2021, one in-person and virtual workshop was organized. The workshop participants were selected based on their roles and responsibilities in policymaking, hepatitis B-related care, PMTCT, and infectious disease research.

### 2.3 Data analysis

Quantitative data were analyzed using SPSS version 2.0, and the results were expressed as frequencies and proportions. Meanwhile, the qualitative data were analyzed thematically by choosing articles and interview questions related to the progress and challenges on hepatitis B elimination strategies, national programmes, standard of care of pregnant women with hepatitis B, and current PMTCT interventions.

### 2.4 Ethical considerations

Ethical approval was obtained from the National Ethics Committee of the Ministry of Health of Cameroon and Zimbabwe, respectively, to carry out the seroprevalence studies. Information from the participants who gave consent to participate in the study were treated with confidentiality. Only the interviewer and data entry staff had access to participants' information that could reveal their identity.

## 3 Results

### 3.1 Composition of the MICHep B network

In 4 years (2018–2022), the Mother–Infant Cohort of Hepatitis B (MICHep B) Network currently includes individuals of different health disciplines and the civil society in Cameroon, Zimbabwe, Gabon, Chad, Central African Republic, and the United Kingdom, and it is coordinated from the Chantal Biya International Reference Center for Research on the Prevention and Management of HIV/AIDS (CIRCB) in Yaoundé, Cameroon. The members of this Network include individuals working in obstetrics/gynecology, pediatrics, gastroenterology, medical research, epidemiology, biostatistics, public health, nursing and midwifery, academia, as well as postgraduates and postdoctoral fellows, and personnel from the Department of Family Health and the Department of Disease Control and Epidemics of the Ministry of Health (MoH). The MICHep B Network is led by a researcher, a lead gynecologist, and a lead pediatrician. From the literature search and presentations during the workshops, these countries reported low hepatitis B vaccination coverage and no existing structured PMTCT of HBV program (Supplementary material 1). Through this network, the members and workshop participants shared the opportunities and challenges, different practices and interventions that could prevent the PMTCT of HBV, and updated their knowledge to incorporate the PMTCT of HBV activities into the successful PMTCT of HIV program. The United Kingdom served as a strong force in the mobilization of funds and training.

### 3.2 In-depth interviews with health care workers in Cameroon

The interview questions were administered to 45 maternity staff, which included midwives, obstetricians, obstetric nurses, and nurse-midwives, MoH staff, and representatives of the gynecology and pediatrics societies in order to understand the importance and challenges of introducing measures for PMTCT of HBV in the maternal and child health services in Cameroon. In particular, their understanding of the use of the hepatitis B birth-dose vaccine and HBIG within 24 h after delivery was sought. Although affordability and accessibility of these PEP products were most frequently mentioned as challenges by the interviewees, most of them reported that families showed the willingness to provide the vaccine and the HBIG to the newborn. A majority of those interviewed showed a high rate of acceptability of the HepB-BD vaccine. In addition, the training curricula, standards, and norms in reproductive health have been revised to include the current, feasible country objective-driven guidelines for the prevention of MTCT of HBV in Cameroon.

### 3.3 HBV serosurvey among pregnant women in Cameroon and Zimbabwe

#### 3.3.1 HBV serosurvey among pregnant women in Cameroon

A hospital-based study was carried out in seven antenatal clinics in health facilities located in rural, semi-urban, or urban settings of the Center Region in Cameroon. At these sites, 1,992 pregnant women were tested for HBsAg. We recorded 115 HBsAg-positive cases (5.8%) and 11 HBV and HIV co-infected (0.6%) cases who were referred to the designated treatment centers. The study showed that 47.6% of pregnant women had never been tested for HBsAg. A total of 31 children were born to the HBV-positive mothers, according to the Yaoundé study in Cameroon, 27 (87.1%) were given hepatitis B birth-dose vaccine, and 25 (80.6%) were given both hepatitis B birth dose and immunoglobulin (HBIG).

#### 3.3.2 Serosurvey of hepatitis B in Zimbabwe

In Harare, Zimbabwe, 1,200 pregnant women attending antenatal care clinics were tested for HBsAg. Among these women, 32 tested positive for HBsAg (2.7%).

### 3.4 Networking and training workshops

A total of two in-person capacity-building and networking workshops were organized in 2018 and 2019 in Yaoundé, Cameroon and one virtual meeting in 2021 due to the COVID-19 pandemic. Overall, 190 participants from Cameroon, Chad, Gabon, Zimbabwe, Central African Republic, the United States, and the UK attended the workshops. The participants included high-ranking MoH personnel, obstetricians/gynecologists, pediatricians, gastroenterologists, medical researchers, epidemiologists, biostatisticians, public health experts, nurses and midwives, and postgraduates and postdoctoral fellows. The workshop participants were selected based on their role in policy-making, hepatitis B-related care, PMTCT, and infectious disease research. However, those who are involved in the implementation of the activities of the MICHep B Network are volunteers receiving no financial remuneration. The workshops organized had more than 60 persons in attendance and more than 130 persons participating online. Platforms for the exchange between policy implementation teams, health personnel, and MoH personnel were created where questions and challenges were channeled to the Family Health Department of the MoH and experts.

These networking workshops aimed at sensitizing the advocates and promoters of the prevention of MTCT of HBV in order to strengthen the existing interventions. The recommendations of the three workshops were (i) to establish networks within countries to promote the PMTCT of hepatitis B; (ii) to promote within each MICHep B Network country, virtual communication tools and other cost-effective ways to disseminate information; (iii) to organize a national forum on PMTCT of hepatitis B; (iv) to improve access to HBV screening of pregnant women and to antiviral agents against hepatitis B; (v) to organize free HBV screening and vaccination against hepatitis B of women of reproductive age; (vi) to raise awareness on the transmission and prevention of HBV in the communities; (vii) to establish strategies and guidelines to follow-up children exposed to HBV through pregnancy; (viii) to carry out operational research on what infant preexposure prophylaxis (PrEP) and maternal antiviral prophylaxis (MAP) to use to mitigate the MTCT of HBV and when to discontinue prophylaxis therapy in case of a co-infection of HBV/HDV, HBV/HIV, and HBV/HCV.

### 3.5 Grant writing workshop and mobilization of funds

In 2018, a workshop on grant writing was carried out in Yaoundé for postgraduate students on the topics “How to Search for Calls of Proposals” and “How to Write a Grant Proposal” with a focus on MTCT and prevention of hepatitis B. In 2018, we obtained the Global Challenges Research Funds Networking grant in collaboration with the University of Oxford, UK. A second grant-writing workshop was organized in 2022 based on the recommendations of the 2021 networking workshop. In 2022, the MICHep B Network received a grant from the Congo Basin Institute Capacity-building Grant to organize a workshop. A total of 30 participants were trained at these workshops.

### 3.6 Capacity-building and training of maternity staff

After the 2019 networking workshop, the lead gynecologist of the MICHep B Network coordinated two meetings of the Ministry of Public Health to revise the (i) norms and standards, procedures, and algorithms in Sexual and Reproductive Health (SRH) to include HBV screening in pregnancy and (ii) pre-service and in-service curricula of Sexual Reproductive Maternal Neonatal Infant Adolescent Health (SRMNIAH). These new guidelines for SRH are used in all hospitals in Cameroon through which a systematic screening of all pregnant women for HBV infection and a birth-dose vaccination of all newborns to HBsAg-positive mothers are promoted.

Furthermore, training of 15 postgraduate students and postdoctoral fellows on various topics were conducted in-person and online. The topics included epidemiology and biostatistics, implementation of hepatitis B birth-dose vaccine, testing of pregnant women for HBV, early infant diagnosis (EID) of HBV infection of children born to HBV-positive mothers, bioethics (online) and good clinical laboratory practice (GCLP), implementation research into policy and practice, scientific plagiarism, writing and publication of scientific articles, systematic reviews, and meta-analysis. The key findings from the survey of the MICHep B Network have been disseminated to key personnel of the MoH. The network is part of the “Birth Dose Vaccine Platform Task Team” working on the project titled “Birth Platform-Strengthening Pilot Study” for the introduction of the hepatitis B birth-dose (HepB-BD) vaccine in the Expanded Programme of Immunization (EPI) in Cameroon.

## 4 Discussion

We sought to set up a multidisciplinary network, the MICHep B Network, with the objective to create awareness among pregnant women, their partners, and the community on PMTCT of HBV and strengthen ongoing strategies to fight against MTCT of HBV.

The MICHepB Network is a footprint initiative of South–South collaboration and North–South collaboration to strengthen programs of global public health importance. There are other advocacy groups in Africa working on the improvement of reproductive health and mother–childcare. These African taskforce or groups include the WHO African Region that launched the Technical Advisory Group on maternal, newborn, child and adolescent health in WHO African Region in 2020, PATH (global health organization) in South Africa, which focuses on improving maternal health, enhancing infant and young child health, and strengthening health systems and disease prevention and treatment efforts (23, 24). Another taskforce that specializes in the fight against hepatitis B is the Coalition for Global Hepatitis Elimination, which partners with Care for Social Welfare International in Cameroon (CASWI), a nonprofit, women and youth development organization, as well as Clinton Health Access Initiative (CHAI), to contribute to the effective use of hepatitis B birth-dose vaccine (25, 26).

However, these initiatives have a common focus of promoting the use of hepatitis B birth-dose vaccination for the prevention of mother-to-child transmission. In most countries in the WHO African Region, there are specific and successful programs for the prevention of vertical transmission of HIV and syphilis, although WHO recommends triple elimination of AIDS, syphilis, and hepatitis B in the mother–infant care continuum. The Coalition for Global Hepatitis Elimination reported in 2022 that 14 African countries have implemented the HepB-BD vaccine in their routine national infant immunization schedule. However, the Hepatitis B-BD vaccine was incorporated in the EPI in Cameroon with evidence and advocacy from the MICHep B Network studies and other research groups (27). In addition, the MICHep B Network enhanced the administration of the hepatitis B birth-dose vaccine in 87.1% and both hepatitis B birth-dose vaccine and immunoglobulin (HBIG) in 80.6% of the children born to the HBV-positive mothers in the cohort in Cameroon. Attaining optimal coverage of Hep B-BD vaccination in these countries is challenging, but at the same time, it is an opportunity to share the experiences, mobilize funds, and support public health systems in the countries of the MICHep B Network fostering a South–South cooperation.

We therefore established the MICHep B Network of multidisciplinary experts from selected countries in Africa to promote cost-effective strategies for PMTCT of HBV. The preliminary activities of the network included a seroprevalence study in Cameroon and Zimbabwe in pregnant women, expanding these activities to other countries with similar challenges and objectives and, finally, organizing capacity-building workshops involving multidisciplinary participants. One of the strengths of MICHep B Network is the support it receives from professional medical groups, the Department of Family Health of the MoH, and a collaborating partner in Europe.

The in-depth interview demonstrated a high rate of acceptability of the use of vaccines, as reported by health care workers and pregnant women. Therefore, supporting activities and efforts of the MICHep B Network in the fight against hepatitis B through the PMTCT of HBV in its member countries could lead to free HBsAg testing of pregnant women, administering hepatitis B birth-dose vaccine and designing a policy on the use of birth dose immunoglobulin.

We recorded a seroprevalence of 5.8% of HBsAg in Cameroon among a population of 1,992 pregnant women in seven health facilities, while the rate was 2.7% in Harare in Zimbabwe among 1,200 pregnant women. The prevalence in Cameroon differs from 6.4% stated by Torimiro et al. (9), 4.98% reported by Fomulu et al. (10), and 10.2% noted by Moutchia et al. (28). The 2.7% of seroprevalence observed in this study among pregnant women in Harare, Zimbabwe is also different from the 13% seroprevalence observed by Dzingirai et al. in Zimbabwe and a similar hospital-based study in Gabon by Kamgaing et al. that showed a rate of 2.4% (11, 29). It is worth noting that the similarities and differences in HBV infection rates between the different MICHep B Network member countries such as have been reported in Chad (12% in the general population vs. 13% among pregnant women), Gabon (10.3% in the general population vs. 9.2% in pregnant women), and Central African Republic (12.6% in the general population vs. 8.2% in pregnant women) (8, 11–13, 30). This finding demonstrates the need for the MICHep B Network to expand to other African countries with similar challenges and opportunities for the prevention of hepatitis B.

In addition, the MICHep B network enhanced the administration of the hepatitis B birth-dose vaccine in 87.1% and both hepatitis B birth-dose vaccine and immunoglobulin (HBIG) in 80.6% of children born to the HBV-positive mothers in the Yaoundé study. This finding is low as compared to the 100% administration of vaccine observed in exposed infants who received both HepB-BD vaccine and HBIG and who were negative in another study in Yaounde (31). These results were different from that reported in Gabon where all exposed children received the hepatitis B vaccine within 24 h but 68.6% of these children did not receive the HBIG because of limited financial resources (32). Among the 1,992 pregnant women tested in Cameroon, 47.6% had never been tested for HBsAg or did not remember having done the test, which is different from that reported in Gabon (84.4%) (32). Of the 1,992 pregnant women, 66.7% were unaware of the hepatitis B vaccine while only 2.9% were vaccinated. These values are lower than the 9.3% hepatitis B vaccination coverage observed in Gabon in pregnant women and 11.7% reported in Yaoundé in Cameroon, but higher than 1.2% and 0.6% observed in the North Region of Cameroon and Yirgalem in Ethiopia, respectively (7, 9, 28, 33, 34). This finding suggests that the low vaccination coverage may be due to limited financial resources and poor knowledge about the transmission and prevention methods of HBV. This finding also suggests that subsidizing the hepatitis B vaccine for the general population would contribute to scaling up the use of the vaccine toward the elimination of hepatitis B.

Another objective was to expand the activities of the MICHep B Network from Cameroon and Zimbabwe to other countries. Currently, the membership has increased to five sub-Saharan countries, namely Cameroon, Chad, Zimbabwe, Gabon, and Central African Republic. The activities of the MICHep B Network have revealed challenges and opportunities in the elimination strategy of hepatitis B by 2030. However, the preliminary findings show that cost-effective and user-friendly services such as virtual communication facilities to inform experts and disseminate information and research findings are of great value to low-medium-income countries (LMICs) in disease control programs.

Therefore, the MICHep B Network serves as a springboard to step up the existing strategies of the governments in Africa for the elimination of hepatitis B through PMTCT programs. We also observed a strong willingness among families in the study sites in Cameroon to pay for the hepatitis B birth-dose vaccine, which indicates that sensitization of the population on the transmission and prevention of hepatitis B and screening of pregnant women for HBsAg would greatly aid in the PMTCT of HBV program in countries where there is no national policy of hepatitis B vaccination at birth.

Finally, the MICHep B Network carried out capacity-building and networking workshops for multidisciplinary participants from different countries from Africa and Europe and the United States. These participants included physicians, students, top-ranking MoH personnel, and the academia who sought policy revision and the amendment of curricula for the education of health care workers on PMTCT while promoting the implementation of WHO recommendations on hepatitis B prevention.

The MICHep B Network strongly recommends the administration of hepatitis B birth-dose vaccine to all newborns. The recommendations from the workshops have been discussed in national and international conferences. The MICHep B Network partners with local advocacy groups and associations such as the gynecology, pediatrics, and gastroenterology societies to strengthen the existing policies in the fight against hepatitis B. The networking workshops created opportunities for the participants to learn and share through a user-friendly and cost-effective platform. The MICHep B Network successfully organized three workshops covering a broad range of topics on hepatitis B, including vaccination, policy, and practice of testing and management of hepatitis B in pregnancy, innovative algorithms for the early diagnosis of HBV infection in children born to HBsAg-positive mothers, and prevention of mother-to-child transmission of HBV. The MICHep B Network has carried out free HBV screening and open awareness campaigns and created cohorts of HBV-positive pregnant women and their infants, as well as co-infected mothers (HBV/HIV and HBV/HDV) and their infants. Three grant applications were submitted, of which two were successful, while training on grant writing of graduate students along with coaching and mentoring is ongoing. Some limitations in achieving the goals of the network include the lockdown experienced during the COVID-19 pandemic and limited funding to organize serosurveys and knowledge, attitude, and practice (KAP) surveys in the member countries. Other challenges faced include a slow rate of policy revision on hepatitis B birth-dose vaccine in some member countries of the network and inadequate human resources to carry out the different activities.

However, the results and recommendations from the networking workshops can be implemented in other countries. It is possible to implement user-friendly, cost-effective in-person and virtual population-based sensitization campaigns in LMIC to accelerate the achievement of WHO's goal of the elimination of hepatitis B by 2030. Networking as in the case of the MICHep B Network enhances South–South cooperation and creates an avenue for individual country disease control initiatives to be scaled up with respect to hepatitis B. This initiative is sustainable and a promising tool in the fight for an HBV-free future. The strength of the MICHep B network study is the opportunity to create a strong collaborative advocacy network that promotes the prevention of mother-to-child transmission of hepatitis B virus in member countries and beyond.

## Data availability statement

The raw data supporting the conclusions of this article will be made available by the authors, without undue reservation.

## Ethics statement

The studies involving humans were approved by National Ethics Committee for Human Health Research. The studies were conducted in accordance with the local legislation and institutional requirements. The participants provided their written informed consent to participate in this study.

## Author contributions

JT: Conceptualization, Data curation, Formal analysis, Funding acquisition, Investigation, Project administration, Supervision, Validation, Writing—original draft, Writing—review & editing. KD: Conceptualization, Data curation, Formal analysis, Funding acquisition, Validation, Writing—review & editing. NG: Data curation, Formal analysis, Methodology, Validation, Writing—review & editing. SMA: Data curation, Formal analysis, Validation, Writing—original draft. JN: Data curation, Formal analysis, Validation, Methodology, Writing—review & editing. CL: Data curation, Methodology, Validation, Writing—review & editing, Project administration. MB: Project administration, Validation, Writing—review & editing, Conceptualization, Formal analysis, Supervision. BN: Formal analysis, Validation, Writing—review & editing, Data curation, Investigation. AM: Data curation, Formal analysis, Validation, Writing—review & editing, Conceptualization, Supervision. CP: Validation, Writing—review & editing, Methodology, Supervision. PA: Formal analysis, Investigation, Methodology, Validation, Writing—review & editing. PP: Formal analysis, Investigation, Methodology, Validation, Writing—review & editing. PD: Formal analysis, Validation, Writing—review & editing, Investigation, Methodology. AF: Validation, Writing—review & editing, Conceptualization, Data curation, Formal analysis. GN: Investigation, Validation, Writing—review & editing, Funding acquisition, Supervision. BW: Methodology, Validation, Writing—review & editing, Formal Analysis, Investigation. RS: Conceptualization, Data curation, Validation, Writing—review & editing, Methodology, Project administration. SA: Conceptualization, Validation, Writing—review & editing, Data curation, Supervision. AG: Conceptualization, Validation, Writing—review & editing. RG: Conceptualization, Supervision, Validation, Writing—review & editing. IF: Conceptualization, Validation, Writing—review & editing, Supervision. EM: Conceptualization, Project administration, Validation, Writing—review & editing. SR-J: Writing—original draft, Conceptualization, Funding acquisition, Project administration, Supervision, Validation. MICHep B Network: Writing—original draft. RM: Conceptualization, Funding acquisition, Methodology, Project administration, Supervision, Validation, Writing—review & editing.
