# Supplementary material for: Toward the elimination of hepatitis B: networking to promote the prevention of vertical transmission of hepatitis B virus through population-based interventions and multidisciplinary groups in Africa
Source: Front Public Health. 2024 Apr 5;12:1283350. doi: 10.3389/fpubh.2024.1283350 (PMC11026850; doi:10.3389/fpubh.2024.1283350)
Supplement: Supplementary file 1 [file Data_Sheet_1.PDF]

Supplement 1: Challenges and opportunities towards the implementation a PMTCT of HBV Programme in the MICHep B Network

CAMEROON

Challenges

- No structured PMTCT of HBV policy/guidelines
- Low rate of hep B vaccination of pregnant women

Opportunities

- Willingness of families to procure hep B BD vaccine and immunoglobulin
- availability of hep B antiviral (Tenofovir), vaccine and immunoglobulin
- Existence of an effective national PMTCT of HIV and Syphilis
- ongoing operational research on HBV in pregnancy

CHAD

Challenges

- Lack of political will
- very weak implementation of national recommendations
- Insufficient health facilities
- Lack of reporting infrastructure and system
- Low maternal involvement in communities
- Scarce or inexistent data on pregnant women
- No systematic HBV screening during pregnancy

Opportunities

- Couple Hep B BD immunization with BCG
- Existence of an Effective national PMTCT of HIV
- Trained community health workers
- availability of hep B antiviral (Tenofovir), GFATM, GAVI support

GABON

Challenges

- No structured PMTCT of HBV policy/guidelines
- No system of sero-surveillance of HBV
- No established program to fight against HBV

Opportunities

- Existence of an effective national PMTCT of HIV and Syphilis
- Incorporated HepB BD in EPI
- Willingness of families to vaccinate new borns

CENTRAL AFRICAN REPUBLIC

Challenges

- Civil war
-No structured PMTCT of HBV policy/guidelines
- No national recommendations
- No established program to fight against HBV

Opportunities

- Existence of an Effective national PMTCT of HIV and Syphilis
- Incorporated HepB BD in the existing EPI vaccine
- Trained community health workers to track pregnancies and administer HepB BD

ZIMBABWE

Challenges

- Lack of national coordination of HBV in pregnant women
- Lack of financial resources
- No formulated policy on HBV screening in pregnant women
- Limited laboratory capacity for serology testing
- Unavailability of antivirals
- No national treatment guidelines for hepatitis B
- Poor knowledge of health personnel and lack of diagnostic kits have led to poor reporting

Opportunities

- Existence of an Effective national PMTCT of HIV and Syphilis
- Operational research: ongoing pilot study on HBV in pregnancy
- Trained community health workers to track pregnancies and administer HepB BD
