# Supplementary material for: Toward the elimination of hepatitis B: networking to promote the prevention of vertical transmission of hepatitis B virus through population-based interventions and multidisciplinary groups in Africa
Source: Front Public Health. 2024 Apr 5;12:1283350. doi: 10.3389/fpubh.2024.1283350 (PMC11026850; doi:10.3389/fpubh.2024.1283350)
Supplement: Supplementary file 2 [file Data_Sheet_2.PDF]

Supplement 2: Flowchart of MICHepB Network activities towards HBV Elimination in Sub-Saharan Africa

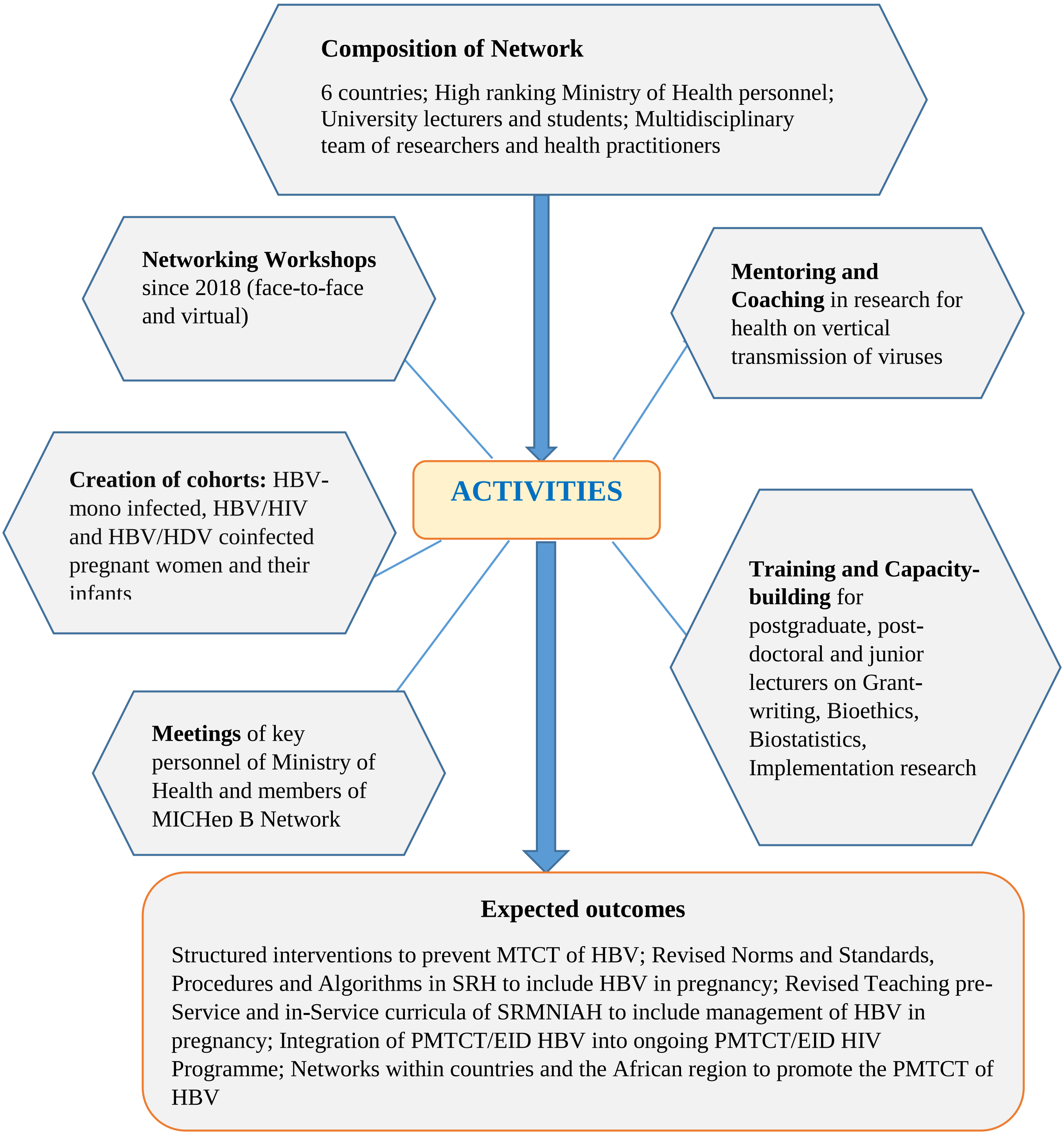

EID: early infant diagnosis; HBV: hepatitis B virus; HDV: hepatitis D virus; HIV: human immunodeficiency virus; PMTCT: prevention of mother to child transmission; SRH: sexual and reproductive health; SRMNIAH: sexual reproductive maternal neonatal infant adolescent health.
